# Exploring the efficacy of deep brain stimulation in pediatric neurological disorders: a comprehensive review

**DOI:** 10.1186/s42494-025-00230-6

**Published:** 2025-07-15

**Authors:** Tianshuang Wang, Xinhua Wang, Yi Wang, Yuanfeng Zhou

**Affiliations:** Department of Neurology, Children’s Hospital of Fudan University, National Children’s Medical Center, No.399 Wanyuan Road, Minhang District, Shanghai, 201102 P. R. China

**Keywords:** Deep brain stimulation, Dystonia, Drug-resistant epilepsy, Tourette syndrome, Neuropsychiatric conditions

## Abstract

Deep brain stimulation (DBS) has emerged as an important therapeutic intervention, effectively addressing a spectrum of drug-resistant neurological and psychiatric disorders. Although its efficacy has been validated in adult populations, the current literature reveals a significant gap concerning its application in pediatric patients. Specifically, pediatric populations afflicted with severe conditions such as dystonia, drug-resistant epilepsy, Tourette syndrome, and some other neuropsychiatric conditions demonstrate an urgent need for alternative therapeutic options. This review systematically examined the existing literature on the application of DBS in pediatric neurological disorders, focusing on the aforementioned conditions. Preliminary findings indicate that while DBS shows potential for a specific subset of pediatric patients, the current data is limited and lacks statistical power. Reported cases exhibit varying degrees of therapeutic success. Although adverse effects associated with DBS in pediatric populations are rare, further investigation is essential to define safety profiles accurately. Future research should focus on conducting large-scale, randomized controlled trials to validate outcomes and determine optimal patient selection criteria, thereby broadening its clinical application within the pediatric population.

Neuromodulation technology encompasses a range of techniques aimed at regulating nervous system activity by stimulating specific areas to treat various diseases. Currently, neuromodulation techniques include deep brain stimulation (DBS), vagus nerve stimulation (VNS), responsive neurostimulation (RNS), and transcranial magnetic stimulation (TMS). Since its inception for the treatment of adult diseases, DBS has demonstrated significant benefits in children with myoclonic dystonia and other conditions. This article reviews advancements in DBS related to pediatric neurological diseases.

## Deep brain stimulation

As an invasive neuromodulation technique, DBS delivers electrical pulses to specific deep structures within the brain to alter or modulate neural function, achieving reversible and adjustable therapeutic or clinically beneficial effects. The effectiveness of DBS is influenced by pathological networks, electrode placement and stimulation parameters, such as pulse width, stimulation interval, frequency, and amplitude. These parameters are typically established during clinical programming and can be adjusted as needed.

## Current indications for DBS in pediatric populations

### Dystonia

Dystonia is a movement disorder marked by sustained or intermittent muscle contractions that lead to abnormal unusual movements and/or postures [1]. DBS for the treatment of pediatric drug-resistant dystonia has been extensively studied and practiced [2–4]. DBS modulates pathological activity through a variety of overlapping mechanisms, including direct suppression of abnormal firing, modulation of neurotransmitter release, alteration of neuronal excitability, and influence on the synchrony of neural networks. In pediatric patients, the globus pallidus internus (GPi) and the subthalamic nucleus (STN) are the most commonly used targets. Currently, there are no data directly comparing GPi and STN stimulation in pediatric dystonia. Previous small-scale and open-label studies suggested that both GPi and STN serve as effective DBS targets with comparable long-term efficacy, as indicated by mean improvements in the Burke-Fahn-Marsden Dystonia Rating Scale (BFMDRS) motor scores: STN-DBS (77–90%) and GPi-DBS (32–72%) [5, 6]. Notably, STN stimulation offers advantages such as rapid onset of action, improvements in ocular and generalized dystonia, and low battery consumption [7, 8]. Nonetheless, targeting the STN in young patients presents challenges, as microelectrode recordings can be less reliable under anesthesia or in the presence of pallidal injuries [9]. Some studies suggested that GPi-DBS may be more effective for patients with axial symptoms [7].

Patients with inherited, isolated, or idiopathic dystonia can achieve long-term improvement in motor function after DBS. A systematic review of 78 pediatric patients who underwent DBS implantation under the age of 21, mostly targeting GPi, demonstrated sustained motor function improvement over an average follow-up period of 8.5 years (up to 22 years), with improvements in the BFMDRS score ranging from 2.5% to 93.2%. During long-term follow-up, no significant cognitive or psychiatric comorbidities were observed [5]. Factors indicating a favorable response to DBS include late-onset age, short disease duration, and axial symptoms in dystonia. Other predictors associated with a good prognosis encompass a lower severity of baseline symptoms, DYT-*TOR1A* mutation, and the absence of skeletal muscle deformities.

Among primary dystonia, the *TOR1A *mutation is the most prevalent genetic cause, and it demonstrates an optimal response to DBS [10, 11]. Specifically, GPi-DBS yielded improvement rates ranging from 42.9% to 100% over a follow-up period spanning from 3 months to 15 years. Conversely, STN-DBS resulted in BFMDRS-MS improvement rates of 70.4% to 90.4%, with early treatment yielding better outcomes, enabling 61% of pediatric patients to discontinue anti-dystonia medications [10]. For DYT-*THAP1* (DYT6), postoperative efficacy was slightly reduced, with BFMDRS-MS improvement rates of 42.2% to 58% with follow-up period of 1–7.7 years. For DYT-*SGCE* (DYT11, myoclonus-dystonia syndrome, [MDS]), both GPi-DBS and thalamic ventral intermediate nucleus (VIM) DBS could alleviate both myoclonic and dystonic symptoms. Notably, GPi-DBS was more effective for dystonia, significantly enhancing the quality of life and social adaptability in pediatric patients [12].

However, for hereditary degenerative and acquired dystonia, the improvement achieved with DBS is relatively modest [5], such as in cerebral palsy (CP) [13–16], neonatal asphyxia, kernicterus, neurodegenerative disorders with brain iron accumulation (NBIA) disorders [17], Lesch-Nyhan Syndrome (LNS) and other diseases. This may be attributed to the severity of the diseases, the age of onset, and the accuracy of clinical scales. A literature review summarized nine pediatric patients with refractory dystonia due to static or progressive brain diseases. These patients had an average age of GPi-DBS implantation of 17 years and an average treatment duration of 84 months and the outcome showed a significant improvement in BFMDRS scores by 19.9% and a long-term quality of life by 28.6% [18].

Koy et al. [13] summarized 14 children with movement disorders following cerebral palsy, with an average age of 14 years. At 24 and 36 months of follow-up, there was significant improvement in the total motor disability score by 34.9 and 32.0 points, respectively, compared to baseline. However, at 36 months, there were no significant changes in BFMDRS motor and disability scores, or quality of life assessments. Correlation analysis indicated that younger patients (aged ≤ 12 years) exhibited more significant improvement in motor disability scores.

The most common type of NBIA disease is pantothenate kinase-associated neurodegeneration (PKAN). A meta-analysis revealed that classic and atypical PKAN had BFMDRS-MS improvement rates of 16% and 45%, respectively [19]. A study summarized 17 pediatric PKAN cases, with an average follow-up period of 13 months after STN-DBS, the BFMDRS score improved by 36 ± 12.9 points, resulting in an improvement rate of 49 ± 8.0% [17]. However, it is worth noting that the BFMDRS scale may not be sufficiently sensitive when assessing complex forms of dystonia and may not fully reflect the overall functional impact on these patients.

LNS can manifest as intense self-mutilating behavior, generalized dystonia, and motor disabilities. The self-mutilating behavior stems from dysfunction of the cortico-basal ganglia-thalamo-cortical circuit, and various basal ganglia subregions may be associated with neural circuits underlying behavioral and motor symptoms. Literature reported on 4 pediatric patients with LNS indicated that, after unilateral/bilateral GPi surgery, all patients exhibited a reduction in self-mutilating behavior. Additionally, two-thirds of the children showed improvement in upper limb function and goal achievement, although there was minimal improvement in dystonia [20]. Salamatova et al. [21] was the first to report a 13-year-old child with autoimmune parkinsonism resulting from influenza A infection. The child did not demonstrate significant improvement following treatment with intravenous immunoglobulin (IVIG), plasmapheresis, and levodopa. However, partial improvements in bradykinesia, tremor, and motor disabilities were observed after bilateral GPi-DBS. While this intervention enhanced the child's disability status and quality of life, it did not halt the progression of the disease.

Additionally, status dystonicus (SD) is a rare movement disorder emergency and it occurs in 60–80% of cases in children or adolescents [22, 23]. It was reported that DBS can also ameliorate symptoms and enhance survival rates in children with SD [4, 5]. A multicenter study and systematic review, which encompassed 85 cases, demonstrated that early DBS surgery and activation (average of 25 days after SD episode, with an average activation at 1.6 days postoperatively, mostly targeting the GPi) could significantly control pediatric symptoms (mostly effective within 2–4 weeks postoperatively). With an average follow-up of 16 months, symptoms improved by 32–51%, and the mortality rate was also significantly lower compared to drug therapy alone (4% vs. 10–12.5%). Furthermore, posterior-ventrolateral GPi stimulation was found to be more effective [4].

### Epilepsy

#### Drug-resistant epilepsy (DRE)

Approximately 30% of children with epilepsy are DRE [24]. For those with bilateral foci, multifocal lesions, or lesions in the dominant hemisphere may not be surgical candidates for resection. At present, the Food and Drug Administration (FDA) has not approved DBS for pediatric epilepsy. While numerous studies have explored the outcomes of DBS in DRE, epilepsy is a clinically heterogeneous disorder with multiple underlying causes. The variability in inclusion criteria and methodologies across published research is particularly notable, especially for pediatric patients. Furthermore, the mechanism of DBS in treating DRE is not yet fully understood. The relevant pathways include the cortico-striatal-thalamic network and the limbic system's Papez circuit. Nuclei located on these neural transmission pathways can serve as targets for electrical stimulation to modulate the transmission of epileptogenic signals [25, 26].

Currently, the most commonly used DBS targets for the treatment of DRE are centromedian thalamic nucleus (CMT) and anterior thalamic nucleus (ANT). A systematic review evaluated 27 studies encompassing 72 pediatric patients (aged 4–18 years) with DRE who underwent DBS. The findings indicated that following a median follow-up period of 14 months, 75% of the patients experienced a reduction in seizure frequency exceeding 50%, while 11% achieved complete seizure freedom postoperatively [27]. Conversely, approximately 15% of the patients did not exhibit any improvement in seizure activity [25]. Univariate analysis suggested that the longer the follow-up period, the greater the likelihood of postoperative seizure control.

Regarding seizure types, for children with generalized seizures, including certain epilepsy syndromes, such as Lennox-Gastaut syndrome (LGS) [28], CMT may be a more effective target, with 80–100% of children achieving favorable outcomes. A systematic review indicated that 80% of LGS patients experienced a reduction in seizure frequency of more than 50%. Among the DBS patients with positive outcomes, all underwent CMT-DBS [27]. The anterolateral region of the CMT has shown better results compared to other areas [27, 29]. However, the ideal CMT target may vary depending on specific syndrome [30]. Conversely, for focal seizures and focal to bilateral tonic-clonic seizures, ANT-DBS demonstrated greater efficacy than CMT-DBS. According to the SANTE trial, stimulating ANT was an effective method for treating frontal or temporal DRE and was approved by the FDA for focal seizures in adults [31, 32]. Specifically, 75% (6/8) of the pediatric patients (aged 14–18 years) experienced a reduction in seizure frequency ranging from 37 to 90% [25]. Furthermore, the anterior portion of the ANT appears to exhibit enhanced effectiveness [33]. Some researchers also explored the clinical efficacy of combined targets therapy for DRE. There was no significant difference in seizure reduction rates between CMT + ANT and CMT alone (60% vs. 56%) [34]. Further follow-up observations are required in this area of research.

In addition to CMT and ANT stimulation, alternative targets have been investigated for DRE, depending upon the localization of the epileptic focus in the patient. Nevertheless, the number of cases is limited, and further studies are needed to determine their efficacy. Bilateral or unilateral hippocampal DBS has emerged as a potential therapeutic target for temporal lobe epilepsy. Reports indicated that 2/5 cases (aged 10–14 years) experienced a 64–80% reduction in seizure frequency following hippocampal DBS [25, 35]. The STN target primarily inhibits the spread of abnormal electrical activity from the substantia nigra, thereby blocking the seizure pathway. Three cases have been reported by bilateral STN target stimulation, one showed a 71.4% reduction in seizures one month postoperatively, while the other two did not experience seizure control [25]. Furthermore, in two cases of hypothalamic hamartoma, unilateral mammillothalamic tract (MMT) DBS treatment resulted in a 86% and 100% reduction in seizures at 13 and 14 months postoperatively with high-frequency stimulation and also improved the children's mood and quality of life [36]. However, the efficacy of these targets remains inconclusive, and the effects on cognitive function, mental state, and neurological outcomes in pediatric populations necessitate further longitudinal studies.

#### Acute phase of epileptic seizures and refractory status epilepticus

DBS can also be applied to cases during the acute phase of epileptic seizures. Sa et al. [37] reported two children (aged 9 and 5 years old) with fever infection related epilepsy syndrome (FIRES) who underwent CMT-DBS on 27th and 37th days of onset, respectively. After the stimulation was activated, the children experienced a notable reduction in clinical and electrical seizures, especially generalized seizures, and the number of seizures increased again after a brief shutdown, further confirming the therapeutic effect of DBS. Another 17-year-old case with super-refractory status epilepticus secondary to immune encephalitis, who underwent CMT-DBS on day 58 of the disease course. Continuous high-frequency DBS significantly improved EEG background activity and reduced the number of daily discharges [38].

### Tourette syndrome

Tourette syndrome (TS) is a chronic and complex neuropsychiatric disorder that typically manifests in childhood or adolescence, characterized by involuntary, sudden, repetitive, and stereotypical motor or vocal tics. While most individuals with TS often presents with frequent and severe episodes, and most affected children can manage their clinical symptoms through education, cognitive-behavioral intervention, and medication. However, a subset of patients remains challenging to treat, significantly impacting their daily life [39]. Furthermore, approximately 5% of children with TS may even exhibit severe self-harming behaviors. In adults with refractory TS, DBS has been found to significantly alleviate symptoms, including reducing the frequency and severity of tics, as well as enhancing psychosocial functioning associated with tics [40–42]. The thalamus and GPi are frequently selected for DBS in adult refractory TS due to their crucial role in motor function regulation [43, 44].

In children with TS, most patients may experience a spontaneous alleviation of symptoms during adolescence, which could complicate the long-term assessment of DBS effectiveness. Considering the potential risks associated with DBS, some experts recommend considering DBS treatment for TS patients over 18 years old [44, 45]. Consequently, the number of pediatric DBS treatments is limited. However, childhood is a crucial period for learning, personality development and psychological well-being, and early symptom improvement may facilitate development and enhance social adaptability.

A meta-analysis of 58 patients with TS (aged 12–21 years) across 21 studies demonstrated a mean postoperative improvement of 57.5% ± 24.6% in the Yale Global Tic Severity Scale (YGTSS) scores, regardless of the targeted brain region. Furthermore, 64% of participants exhibited an enhancement of more than 50% in their postoperative scores [46]. Another review study indicated that the improvement in tic symptoms following DBS gradually emerges over time, with a median duration of 13 months to achieve a 40% reduction in tics. This implies that significant clinical improvement may require approximately one year of follow-up after DBS treatment in children [47].

Identifying the optimal brain target for an individual's symptom profile remains challenging. The most frequently utilized targets were in the medial thalamic region. In patients with milder symptoms of TS, thalamic stimulation showed more significant improvement compared to GPi target [46]. GPi-DBS has been demonstrated to significantly reduce the severity of both motor and vocal tics, as well as depressive symptoms associated with TS [48]. Approximately 70% of cases exhibited a reduction exceeding 50% in YGTSS scores [49]. Another study corroborated these findings, reporting an average reduction of 48% in motor tics and 56.5% in vocal tics among 11 patients [50].

However, the optimal parameters for effective stimulation remain indeterminate. While some studies suggested that increased stimulation pulse widths correlated with improved outcomes, others have not consistently observed this relationship [46]. Stimulation parameters probably require thorough evaluation and individualized adjustment to optimize therapeutic efficacy.

### Other neuropsychiatric conditions

DBS is increasingly being utilized in the treatment of various disorders, such as drug-resistant depression, obsessive-compulsive disorder (OCD), anorexia nervosa, anxiety, and schizophrenia in adults [51]. However, some of these disorders, like OCD, may spontaneously remit by early adulthood, which explains the limited number of cases of DBS treatment in the pediatric population. A systematic review summarized nine cases of extreme behaviors related to autism spectrum disorder (ASD), including four children aged 13–17 with targets such as the basolateral amygdala, GPi, posterior hypothalamus (pHyp), and nucleus accumbens (NAcc). Postoperative symptom assessments demonstrated varying degrees of improvement [52]. For patients with anorexia nervosa, the primary DBS targets are the subcallosal cingulate (SCC) and NAcc, with an average BMI increase of 24.82% over 17.1 months. In children, NAcc-DBS has shown reduced hypermetabolism in certain brain areas, indicating its potential role in regulating abnormal brain metabolism [53].

## Safety of DBS

**Fig. 1:**
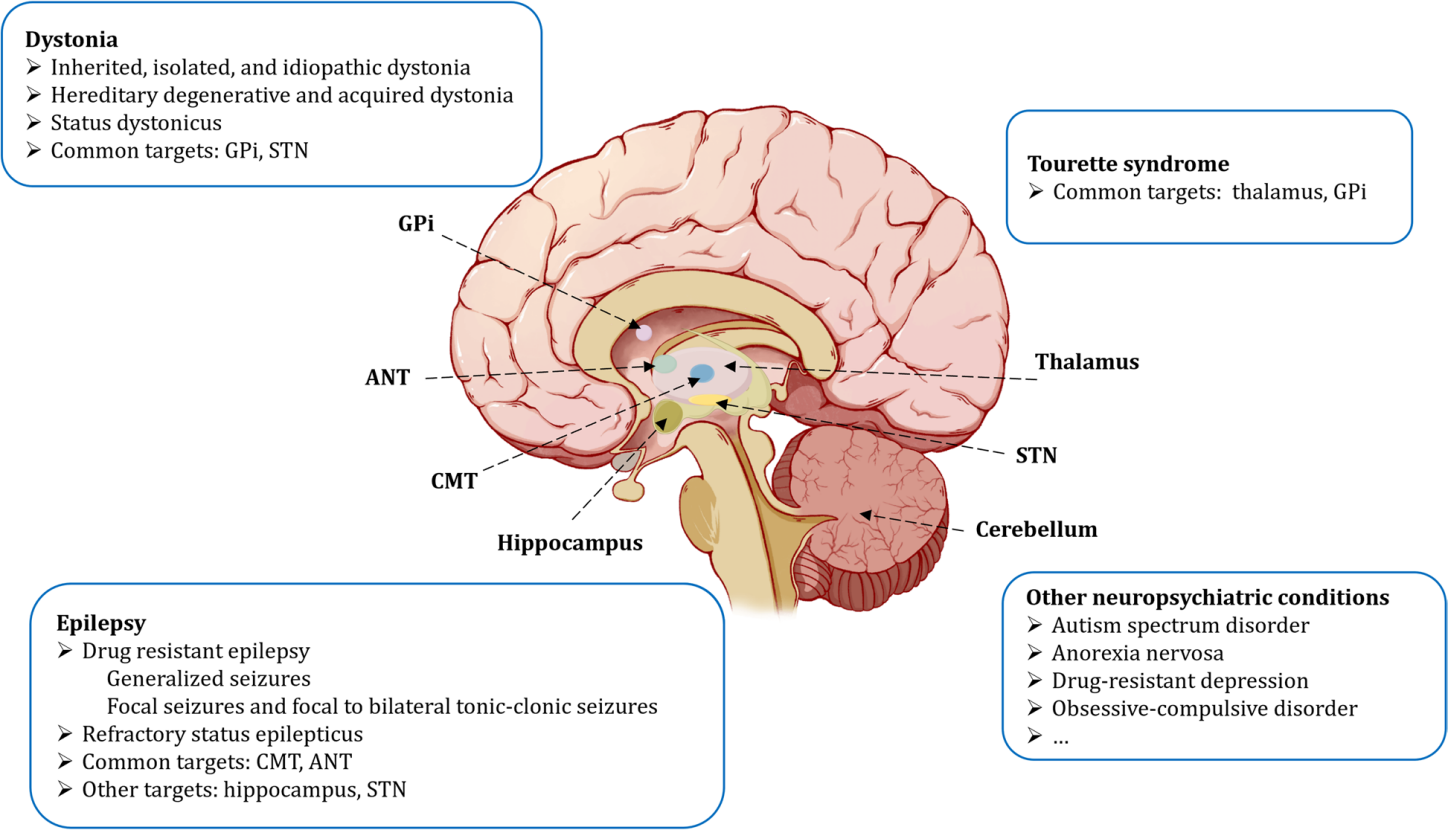
Common targets for DBS in treatment of pediatric neurological disorders. Abbreviations: DBS, deep brain stimulation; GPi, globus pallidus internus; STN, subthalamic nucleus; CMT, centromedian thalamic nucleus; ANT, anterior thalamic nucleus

The safety and complication management strategies of DBS involve multiple aspects, including preoperative assessment, intraoperative safety measures, and postoperative complication management and prevention [54]. Generally, DBS surgery has minimal side effects. A review of 13 DBS clinical trials in pediatric populations with 63 participants reported 18 adverse events, including post-operative infection (22.2%), headache (11.1%), and hemorrhage (5.6%) [55]. Hardware-related complications may involve lead migration, fracture or disconnection of the connecting wire, as well as malfunction of the neurostimulator. There was one reported case of electrode lead fracture occurring 31 months post-surgery, but the therapeutic effect on seizures remained unaffected following the replacement of the battery and electrode [56]. Additionally, some children exhibited excessive emotion and aggressive behavior postoperatively, which could be reversed by turning off the DBS stimulator [47]. Given the continuous growth and development in children, pediatric patients often require more frequent parameter adjustments to optimize clinical needs compared to adults [57]. To enhance the efficacy of DBS and mitigate the issue of battery depletion, responsive DBS technology has been developed which can dynamically adjust stimulation based on the clinical symptoms, thereby improving the efficiency of treatment and the patient's quality of life Fig. 1.

## Conclusion

In conclusion, pediatric neurological disorders represent a heterogeneous group of conditions. Studies have provided evidence that DBS is a potentially potent treatment for pediatric patients. It is imperative that patients should undergo thorough evaluation and selection process by a multidisciplinary team to optimize treatment efficacy. While several effective targets have been identified for specific disorders, these targets are associated with varying therapeutic outcomes. Nevertheless, the optimal clinical target or combination of targets has yet to be definitively established. Comprehensive and detailed documentation of all surgical and programming procedures is essential. Furthermore, there is a need for more large-scale randomized controlled trials with larger sample sizes to determine the optimal localization and stimulation parameters, as well as to broaden the analysis of predictive factors for clinical response. Unlike adult patients, developmental and psychosocial factors should also be considered in pediatric populations. A key objective of future studies on DBS treatment is to enhance clinical symptoms while simultaneously improving functional impairments and quality of life.

## Data Availability

Not applicable.

## Abbreviations

ANT: Anterior thalamic nucleus
ASD: Autism spectrum disorder
CMT: Centromedian thalamic nucleus
CP: Cerebral palsy
DRE: Drug-resistant epilepsy
DBS: Deep brain stimulation
FIRES: Fever infection related epilepsy syndrome
GPi: Globus pallidus internus
IVIG: Intravenous immunoglobulin
LGS: Lennox-Gastaut syndrome
LNS: Lesch-Nyhan Syndrome
NBIA: Neurodegenerative disorders with brain iron accumulation
OCD: Obsessive-compulsive disorder
PKAN: Pantothenate kinase associated neurodegeneration
RNS: Responsive neurostimulation
SCC: Subcallosal cingulate
SD: Status dystonicus
STN: Subthalamic nucleus
TS: Tourette syndrome
TMS: Transcranial magnetic stimulation
VIM: Thalamic ventral intermediate nucleus
VNS: Vagus nerve stimulation
